# Unveiling cancer dormancy: Intrinsic mechanisms and extrinsic forces

**DOI:** 10.1016/j.canlet.2024.216899

**Published:** 2024-04-21

**Authors:** Ruihua Liu, Yawei Zhao, Shang Su, Augustine Kwabil, Prisca Chinonso Njoku, Haiquan Yu, Xiaohong Li

**Affiliations:** a School of Life Sciences, Inner Mongolia University, Hohhot, Inner Mongolia Autonomous Region, 010070, China; b Department of Cell and Cancer Biology, College of Medicine and Life Sciences, The University of Toledo, Toledo, OH, 43614, USA

**Keywords:** Disseminated tumor cells, Dormancy markers, Dormancy niches, Self-seeding, Relapse/recurrence

## Abstract

Tumor cells disseminate in various distant organs at early stages of cancer progression. These disseminated tumor cells (DTCs) can stay dormant/quiescent without causing patient symptoms for years or decades. These dormant tumor cells survive despite curative treatments by entering growth arrest, escaping immune surveillance, and/or developing drug resistance. However, these dormant cells can reactivate to proliferate, causing metastatic progression and/or relapse, posing a threat to patients’ survival. It’s unclear how cancer cells maintain dormancy and what triggers their reactivation. What are better approaches to prevent metastatic progression and relapse through harnessing cancer dormancy? To answer these remaining questions, we reviewed the studies of tumor dormancy and reactivation in various types of cancer using different model systems, including the brief history of dormancy studies, the intrinsic characteristics of dormant cells, and the external cues at the cellular and molecular levels. Furthermore, we discussed future directions in the field and the strategies for manipulating dormancy to prevent metastatic progression and recurrence.

## 1. Introduction

Metastases and cancer relapse are the major causes of cancer death [1-8]. In developed countries, most cancer patients were diagnosed at the early stage without metastases. However, metastases developed, and cancer relapsed years or decades later despite lines of standard care [9]. Based on clinical observations and preclinical studies, these metastatic progression and relapse are likely caused by the reactivation of early disseminated tumor cells (DTCs) that are dormant initially. In preclinical models, studies have uncovered the dormancy at the cellular and tumor mass levels, revealed the dormancy niches, and demonstrated factors that reactivate or induce dormancy [10]. In the clinic, DTCs were identified in the patient’s bone marrow or lymph nodes, and some dormant cell gene expression correlations in preclinical models with patient data were reported [11-15]. However, we still face enormous challenges and barriers to achieving successful clinical benefits in manipulating the DTCs, i.e., eliminating them or maintaining the DTCs dormant to reach an operational cure.

This review aims to delineate the future research prospects and clinical translational potential in the context of cancer dormancy. We provide an overview of the current understanding of tumor dormancy and reactivation, including the identities of the dormant DTCs, their interaction with the microenvironment, and the diverse experimental models employed in dormancy investigations. Furthermore, we discuss the potential strategies to manipulate cancer dormancy to mitigate metastatic progression and prevent cancer recurrence.

## 2. The brief history of cancer dormancy

In 1934, Willis first used the term “dormancy” to explain the phenomenon of delayed metastasis in cancer patients who showed no signs of local recurrence [16]. In 1954, the British Medical Journal reported that Dr. Geoffrey Hadfield first used “dormant” to replace the word “latent,” which has been utilized to describe the exceptions that the growth of malignant tumors was not continuous from beginning to end in clinical cases [17]. This short article justified using “dormant” because it suggests cancer cells’ resting or sleeping phase. Spontaneous regression of malignant tumors with speculations about dormancy in the clinic was documented two years later [18]. Experimentally, Science (1959) reported an elegant and pioneering study by Drs. Fisher and Fisher provided direct evidence of dormancy. Fifty Walker-256 carcinoma cells were injected intraportally into rats. Three months after injection, one hundred percent had a tumor within a few weeks only in rats were subjected to repeated laparotomy. However, none was found in the ones without laparotomy [19]. Another study validated this traumatic stimulus of dormant tumor growth through liver massage [20]. Together, these studies suggested that the traumatic stimulus recruits circulating cells and that hepatic regeneration is needed to activate the dormant cells. Thus, cancer dormancy recalls not only the latency of cancer patients but also astonishing experimental phenomena at that time, such as the survival of cancer cells from frozen animal tumor tissue [17,21]. Tumor dormancy observations and experiments were gathered and documented at this early stage.

At this early stage, dormancy is still considered a temporary growth arrest. Since 1970, mechanistic studies have been drawn to prove cancer dormancy. Dr. Folkman’s studies further extended the concept of dormancy from non-proliferative cells to a balance between cell proliferation and death. He first used “population dormancy” to describe the dormant status of a small mass of tumor cells. In a rat model, he established the association between dormancy and the prevention of neovascularization [22]. Dr. Folkman and colleagues were also the first to demonstrate proliferation arrest and reactivation of cancer dormancy [23]. They found that cancer spheroids on soft agar reached growth arrest after linear growth. Thymidine incorporation assay showed that the proliferated cells were on the surface of the spheroids. The number of labeled cells, i.e., the proliferating cells, gradually reduced until they approached the dormant size and occupied only a single layer on the outmost rim of the spheroid. When the dormant spheroids were pulsed with [^3^H]thymidine for 7–24 h and cultured for seven days in a fresh medium, labeled cells were found in the middle of the spheroids but not in the outer zone. These dormant cancer spheroids can be maintained for up to one hundred days. During these times, new spheroids or monolayer cultures could be produced by cells shed or isolated from the dormant spheroids. Continuing the group’s focus on angiogenesis, they showed angiogenesis inhibition through angiostatin suppressed primary tumor growth and prolonged metastatic dormancy by inducing a balance between tumor cell proliferation and apoptosis [22,24,25]. The angiogenic switch is a process by which tumors develop new blood vessels. If tumors fail to trigger this angiogenic switch, they will remain dormant. Otherwise, angiogenesis switches dormancy to overt growth/metastases [26-29]. These pioneering studies led to the definition of tumor mass dormancy, i.e., a balance of tumor cell growth and death. Another study investigated cancer dormancy at the cellular level. It revealed that the dormant B cell lymphoma cells were arrested at the G_1_ phase due to inhibiting the cyclin E-cyclin-dependent kinase 2 (CDK2) complex with an induction of p21 [30].

The cancer dormancy studies continued to be challenging and exciting. Based on the definitions of tumor mass dormancy and cellular dormancy, studies have revealed profiles of the dormant cells, the intrinsic and extrinsic signaling affecting dormancy and reactivation, and the dormancy niches, i.e., which organs support dormant cancer cells and how the cancer cells maintain dormant through cell-cell, and cell-extracellular matrix interactions. Intrinsic markers and signatures include the ratio of P38^high^/Extracellular signal-regulated kinase (ERK)^low^ and Nuclear Receptor Subfamily 2 Group F Member 1 (NR2F1) in multiple cancers by Dr. Aguirre-Ghiso and colleagues [31-34], Axl and the transcriptome signature of mitochondrial-related signaling and biological processes in prostate cancer (PCa) cells [35-40]. PCa cells compete with hematopoietic stem cells (HSCs) for the niche in the bone, as demonstrated by Dr. Russell Taichman and colleagues [13,15,41-44]. The PCa cells survived in the HSC niche by adhering to the osteoblasts via Annexin II mediated adhesion complex. Other groups, including ours, demonstrated the osteoblast’s induction of dormancy in PCa through secreted factors or focal adhesion kinase (FAK) inhibition and in multiple myeloma (MM) through the inductions of immune-response genes and genes associated with myeloid cell differentiation [40,45-47]. Studies in breast cancer (BCa) showed that the perivascular niche maintained BCa cell dormancy through integrin β_1_ and/or α_v_β_3_, thrombospondin-1, or E-selectin [14,48,49]. On the other hand, studies from Xiang H.-F. Zhang’s group demonstrated that the osteogenic niche promoted BCa colonization and boosted tumor proliferation via adherens junctions (AJs) and connexin 43 mediated gap junctions formed between osteogenic cells and BCa cells, which further induced the downstream mammalian target of rapamycin (mTOR) and calcium pathways, respectively [50,51]. Notably, “self-seeding” theory has been overlooked, i.e., seeding not only to regional and distant sites but also returning to the tumor itself, developed from pure speculation to experimental demonstrations [52,53]. We will discuss this further in the following section.

**Fig. 1.**
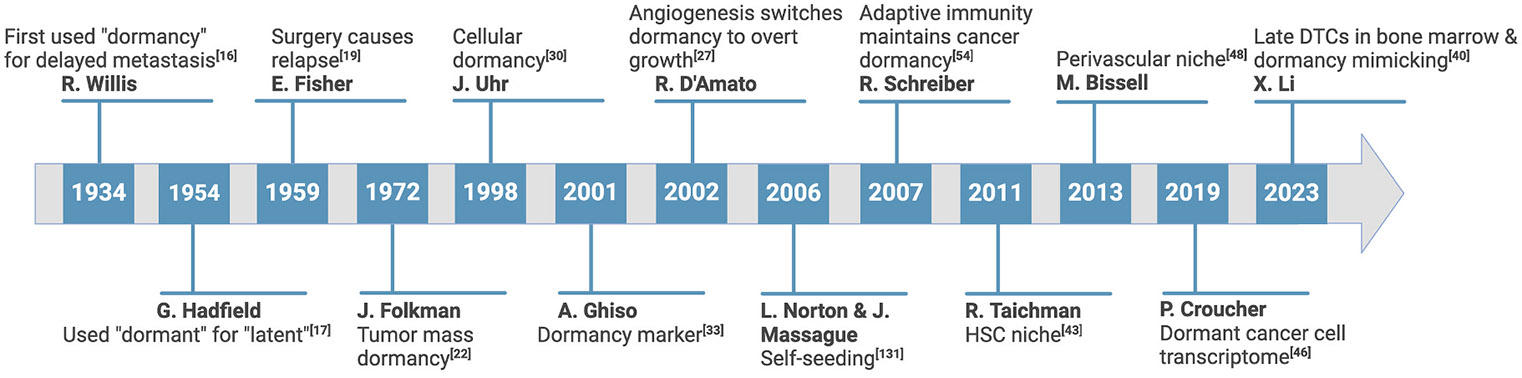
Brief history of cancer dormancy discoveries. The timeline shows the original and impactful discoveries and studies in cancer dormancy both in the clinic and labs. These important studies contributed to our knowledge of angiogenesis switches, immune surveillance, dormancy markers/transcriptome, niches, late dissemination in the bone marrow than the bone cortex, and dormancy-mimicking approach.

Distinct from the immune system’s capacity to destroy cancer, adaptive immunity could maintain the dormant state of occult tumors induced by a chemical carcinogen, in a study elegantly demonstrated by Dr. Robert Schreiber and colleagues [54]. Note that the early studies on the immune system causing tumor dormancy date back to the 1980s [55]. Furthermore, our recent study showed, for the first time, that DTCs were eliminated, i.e., not detectable, in any organs except the bones after early tumor removal in a mouse model and revealed “dormancy mimicking” as a potential translational approach to prevent relapse and metastatic progression [40]. These representative discoveries and studies are summarized in Fig. 1 and will be further discussed in the following sections.

## 3. The dormancy markers and signatures: what are the dormant cancer cells?

Tumor cell dormancy refers to the reversible quiescent state, where the cell cycle arrests at the G_0_/G_1_ phase [30]. In this state, dormant cells survive detection and escape chemotherapy and immune surveillance. Once reactivated, these cells can proliferate quickly to a tumor mass and overt metastases. Senescent tumor cells, cancer stem/pluripotent cells, or cancer-initiating cells can also undergo cell cycle arrest. However, canonical senescent cells have irreversible cell cycle arrest and increased lysosomal senescence-associated β-galactosidase activity [56]; cancer stem/pluripotent, or cancer-initiating cells are cells on top of the lineage hierarchy. Once activated, they divide to give rise to the daughter cells. The detailed differences between cancer stem cells and dormant cells were well summarized in a recent review by Dr. Peter Croucher [10]. The functional definition of dormancy, i.e., the reversible proliferation ability, is well-accepted in the field. Extended efforts have been invested in identifying dormant cancer cells’ expression markers and profiles. These markers, regulations, and references are summarized in Table 1. In the early studies, the readouts and validations of these dormancy markers and gene expression signatures rely on the measurement and comparisons of the tumorigenicity and metastases. In recent developments, DTCs have been the focus. Also noted, correlations with patient data and clinical sample validations are essential for the significance of the identified markers and signatures.

Among these studies, the down-regulation of urokinase plasminogen activator receptor (uPAR), using its 5’ fragment of antisense complimentary (c-DNA), was the first to be implied as a dormancy marker. The uPAR down-regulation was revealed in Hep3, a highly malignant human epidermoid carcinoma cell, either in the chick embryo chorioallantoic membrane (CAM) model or in mice with reduced invasiveness and tumor latency. uPA/uPAR physically associates with integrin α_v_β_1_. Low uPAR led to the reduction of integrin α_v_β_1_ and fibronectin (FN), resulting in ERK1/2 upregulation and activation [31-33,57-59]. P38α/β^High^ERK^Low^ and NR2F1 are the dormancy markers observed in many cancers in respective preclinical models and correlated with patient data [32,34,45,60-67]. Some markers are more specific to a particular type of cancer or organ-specific metastatic recurrence, such as Axl for androgen receptor (AR)-negative PCa and melanoma [35,37-39,68,69] and NKG2D ligand (NKG2DL) in cerebral metastatic BCa and lung cancer [83]. Various markers have been identified for BCa, potentially due to the advanced research in the field and the diverse subtypes of BCa. On the other hand, transcriptome signatures might be more accurate and logical in defining the state of dormant cancer cells. For example, the down-regulated mTOR and calcium signaling pathway in BCa [50,51] and, most recently, the downregulations in mitochondrial-related biological processes in dormant AR-positive PCa cells, an outcome of the dormant PCa cells in bone microenvironment regulated by FAK phosphorylation inhibition [40].

Altogether, our take-home messages from these studies are: 1) The dormancy markers are diverse and context-dependent, reminiscent of the various cancer types and heterogeneity of the tumor cells. For example, P38^High^/ERK^Low^ has been identified as a dormancy marker in many cancers, from the models of CAM to the correlations in patient data. However, opposite results or no change were reported in dormant cancer cells from non-small cell lung cancer [84], Bone morphogenetic protein 1 (BMP1)-induced and osteoblasts-induced PCa dormancy [40,85], and dormant T acute lymphoblastic leukemia (T-ALL) cells after Notch3 inactivation [86]. NR2F1 is a well-recognized dormancy marker, and the protein level is reversely correlated with metastases and recurrence in many cancers, including BCa; a recent paper found that NR2F1 is expressed predominantly in cancer-associated fibroblasts [87]. Cancer cells progress and develop therapy resistance under hypoxia conditions of the primary tumors and some metastatic organs such as bones and brains, at least in part, through an increase in the expression of HIF-1α [88-90]. However, for some cancer cells and DTCs, the hypoxia conditions provided an oasis of dormancy through upregulating HIF-1α; thus, the increased HIF-1α expression in cancer cells is a double-edged sword for cancer cell survival through different downstream mediators [75-80]. Therefore, it’s important to delineate the identities of the cancer cells that responded differently and downstream mediators. 2) Markers are not necessarily the driver of the dormancy. For example, Dr. Taichman’s group found that knockdown of MER Proto-Oncogene, Tyrosine Kinase (MERTK), but not the marker AXL, induced PCa dormancy [68]. Our unpublished data also showed that although NR2F1 was increased as a marker of cell dormancy, knockdown or overexpression NR2F1 did not change the dormancy status or induce dormancy of PCa. 3) Recognizing the limitations of dormancy studies is highly appreciated. New markers and gene/protein expression signatures will be revealed, and the previous ones will be further characterized, validated, and defined. Developing robust readouts, model systems, and clinical validations are essential to overcoming the limitations.

## 4. The dormancy niches: where and how do the cells hide?

In the developed world, most cancer patients (except fifty percent of pancreatic cancer patients who were diagnosed with metastases) were diagnosed without metastases. However, metastases developed despite lines of standard care, including primary tumor removal, chemotherapy, and/or precision treatment, and accounted for the majority of cancer deaths [9]. Since the Seed and Soil theory, it’s generally believed that cancer cells seed at early stages in various distant organs, i.e., disseminated tumor cells (DTCs), are the origins of the metastases, at least partly [91,92]. Therefore, it’s essential to understand where the early and dormant DTCs reside and the interactions of the DTCs with the resident microenvironment at the cellular and molecular levels, i.e., the dormancy niches. These DTCs can be initially dormant at either cellular and/or tumor mass levels and asymptomatic, thus avoiding detection, existing immune attacks, and escaping therapies. The reactivation of these dormant DTCs eventually causes overt metastases and recurrence. In this situation, the dormancy niches are the metastatic niches regarding location. We will focus on and discuss the distinct organ-specific features of dormancy niches because the organ-specific dormancy niches have not been systemically reviewed. We will also examine the uncommon Self-seeding theory.

### 4.1. Liver: the non-parenchymal cells, hepatocytes, and extracellular matrix (ECM)

**Fig. 2.**
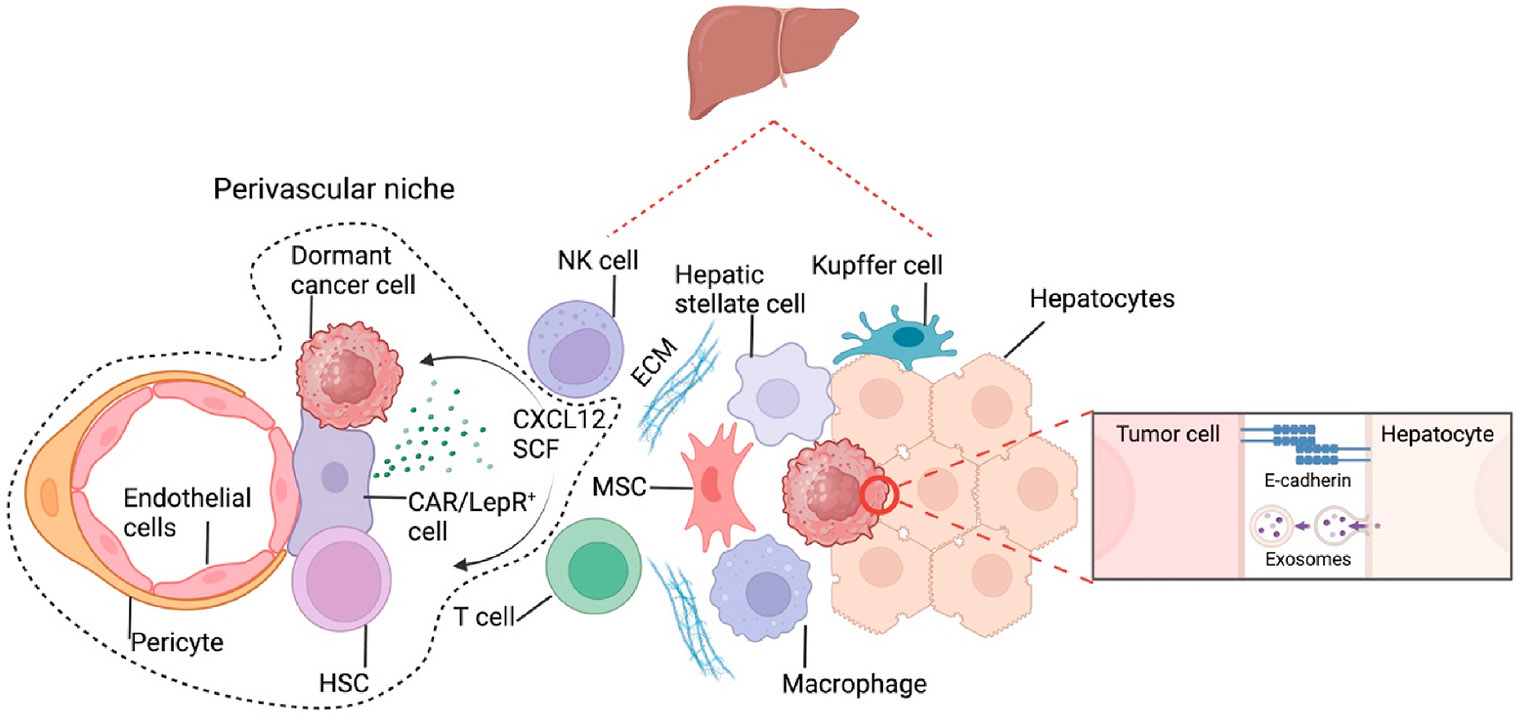
Dormancy niches in the liver. Cancer cells have been found in the perivascular niches of the liver, where hematopoietic stem cells (HSCs) are usually found. Dormancy niches in the liver mainly comprise non-parenchymal cells (NPCs), including immune cells, stromal cells, endothelial cells, and hepatic stellate cells. These NPCs regulate cancer cells’ dormancy through physical contact and secreted factors. Although parenchymal cells, such as hepatocytes, are not part of the niches, they can still affect the fate of cancer cells through cell-cell interactions or exosomes. HSC: Hematopoietic stem cell; CXCL12: CXC motif chemokine ligand 12; CAR cell: CXCL12-abundant reticular cell; LepR: Leptin receptor; SCF: Stem cell factor; MSC: Mesenchymal stem/stromal cell; ECM: Extracellular matrix; NK cell: Natural killer cell.

The liver is one of the most common sites of cancer metastases in most cancers [93]. Liver metastases cause nearly half of the deaths from solid tumors [94]. Early studies in rats demonstrated that traumatic stimulus recruits circulating cells and that hepatic regeneration activates the dormant cancer cells [19,20]. Alan Wells and colleagues reconstituted the non-parenchymal cells (NPCs) comprising endothelial, stromal, and immune cells and hepatocytes to form an *ex vivo* hepatic microphysiologic system. They found spontaneous growth attenuation of a sub-population of BCa cells seed on this system. Therefore, it’s possible a dormancy or metastatic niche that recapitulates the *in vivo* situation [95,96]. Follow-up studies from this group revealed that secret factors from the NPCs, the ratio of NPCs with BCa cells, and inflammatory cytokines such as IL-8 secreted from stellate cells contributed to the escape of dormancy and outgrowth of BCa cells [97,98]. Hepatocytes could facilitate the cancer cell seeding, induce dormancy through exosomes, or contribute to the outgrowth and overt metastases through direct contact to induce E-cadherin expression of cancer cells (Fig. 2) [99,100]. It’s highly anticipated that follow-up studies using this system will further delineate the specific NPCs or interactions among NPCs and cancer cells and profile the cancer cells that can be induced to dormancy and whether and how the dormant cancer cells can be reactivated [101]. Furthermore, hepatic stellate cells secreted CXC motif chemokine ligand 12 (CXCL12), which binds its receptor, CXC motif chemokine receptor 4 (CXCR4), in natural killer (NK) cells, reverses BCa dormancy induced by NK cells through interferon-g [102]. Some hepatic stellate cells synthesize and degrade the ECM. When the liver is damaged, hepatic stellate cells produce large amounts of collagen, a major cause of liver fibrosis. ECM, or a fibrotic environment, has been demonstrated to induce metastatic growth from the dormant cells [103-106].

### 4.2. Bone: the essential of HSC niches

**Fig. 3.**
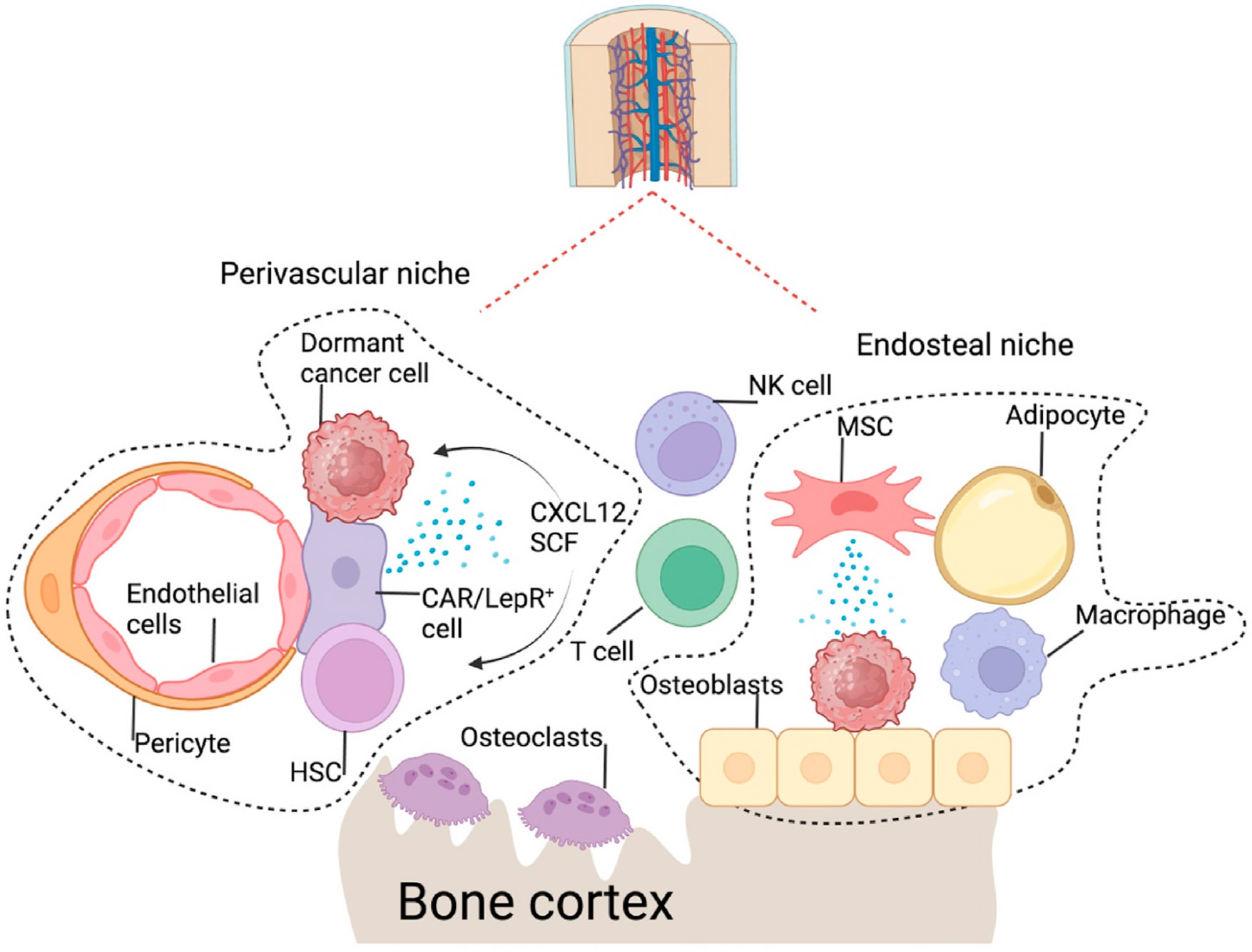
Dormancy niches in bone. Disseminated tumor cells (DTCs) can be found in various niches in the bone, including the perivascular and endosteal niches meant for hematopoietic stem cells (HSCs). The perivascular niches consist of endothelial cells, pericytes, and CAR/LepR-positive cells. In contrast, the endosteal niches on the endosteal surface involve osteoblasts, mesenchymal stem/stromal cells, adipocytes, and macrophages. Notably, osteoclasts are not part of either niche but play a crucial role in creating a growth-suppressive environment. HSC: Hematopoietic stem cell; CXCL12: CXC motif chemokine ligand 12; CAR cell: CXCL12-abundant reticular cell; LepR: Leptin receptor; SCF: Stem cell factor; MSC: Mesenchymal stem/stromal cell; NK cell: Natural killer cell.

The bone is one of the most significant organs for dormancy and metastases and was speculated as the first metastatic site for secondary dissemination [107-109]. At the local bone microenvironment, the HSC niche can be occupied by PC3 PCa or by myeloma cells to maintain dormancy [13,15,41,42]. Osteolineage cells such as osteoblasts are demonstrated in many groups’ studies as the essential cells for the niche where cancer cells are directly localized or within proximity through physical contact or paracrine factors, respectively [40,45-47]. Many cells, including osteoblasts, stromal cells in the perivascular area, peri-sinusoidal, peri-arteriolar, endothelial cells, nerve cells, and adipocytes, were shown to be involved (Fig. 3); the cellular composition of the HSC niche is still controversial and evolving [110,111]. Therefore, further investigation is warranted to determine the interactions of dormant cancer cells with the HSC niche. In BCa, studies showed that the dormant niche consisted of perivascular/sinusoidal stromal cells [14,48,49,112]. Furthermore, other bone resident cells, such as osteoclasts, were shown to awaken the dormant cells in the niches [47,113]. Clinically, DTCs were detected in the patient’s bone marrow of 30 % BCa and 70 % PCa at times of surgery [11,114-117]. Considering the detection of DTCs from the 1–5 mL of bone marrow samples from an organ of approximately 3 L total volume, it is likely a much higher percentage of patients harbor DTCs in the bone marrow [118]. Furthermore, our recent pre-clinical study showed that PCa DTCs were detected in the bone cortex at least six weeks earlier than the detection from the bone marrow. After tumor removal, no DTCs were detected in other organs except bones; the bone marrow DTCs could not be detected until ten weeks later than the bone DTCs [40]. Considering the clinical observations and the preclinical data, we speculated that DTCs in the patient’s bone marrow are the ones that migrated from the bone cortex and are likely to proliferate and eventually develop into overt bone metastases. Furthermore, compared to the lung, bone marrow was shown to be a more dormancy-permissive environment for a head and neck squamous cell carcinoma (HNSCC) model [61].

### 4.3. Lung: no consensus on the dormancy niche is reached

Mina Bissell and her group have demonstrated that the basement membrane, such as laminin-111, constitutes a dormancy niche for BCa cells [119]. Logically chasing the hematogenous route of cancer metastases *in vivo*, they first identified the cells in the perivascular area, i.e., the endothelial cells, being an essential player for the dormancy niche in many organs, including the lung [48].

Models and tools are essential in studying cancer dormancy—for example, D2A1 and D2.0R cells are murine mammary tumor cell lines from the same precancerous D2 hyperplastic alveolar nodule (HAN) line. However, D2A1 is fast-growing and D2. 0R cells can maintain solitary dormancy after metastasizing in a particular organ or tissue, such as the lung, and are independent of the adaptive immune system [120-123]. Using these well-characterized proliferative and dormant cell lines, Erik Sahai and colleagues discovered that the dormant cells interacted with the alveolar epithelial type I cells, which induced D2.0R cells’ expression of the secreted frizzled-related protein 2 (SFRP2). In turn, SFRP2 promoted the organization of fibronectin into fibril. This study demonstrated the involvement of the alveolar epithelial type I cells and subsequent fibril formation of the dormancy niche in the lung. However, the apparent limitation of the studies generated using these two cell lines is the lack of repetitions using other sets of cell lines or human cells. Similarly, a recent study using 4T1 and its dormant derivative, 4T07, showed that CD39^+^PD-1^+^CD4^+^ T cells are necessary and sufficient for 4T07 cells to maintain dormancy in the lung [124].

**Fig. 4.**
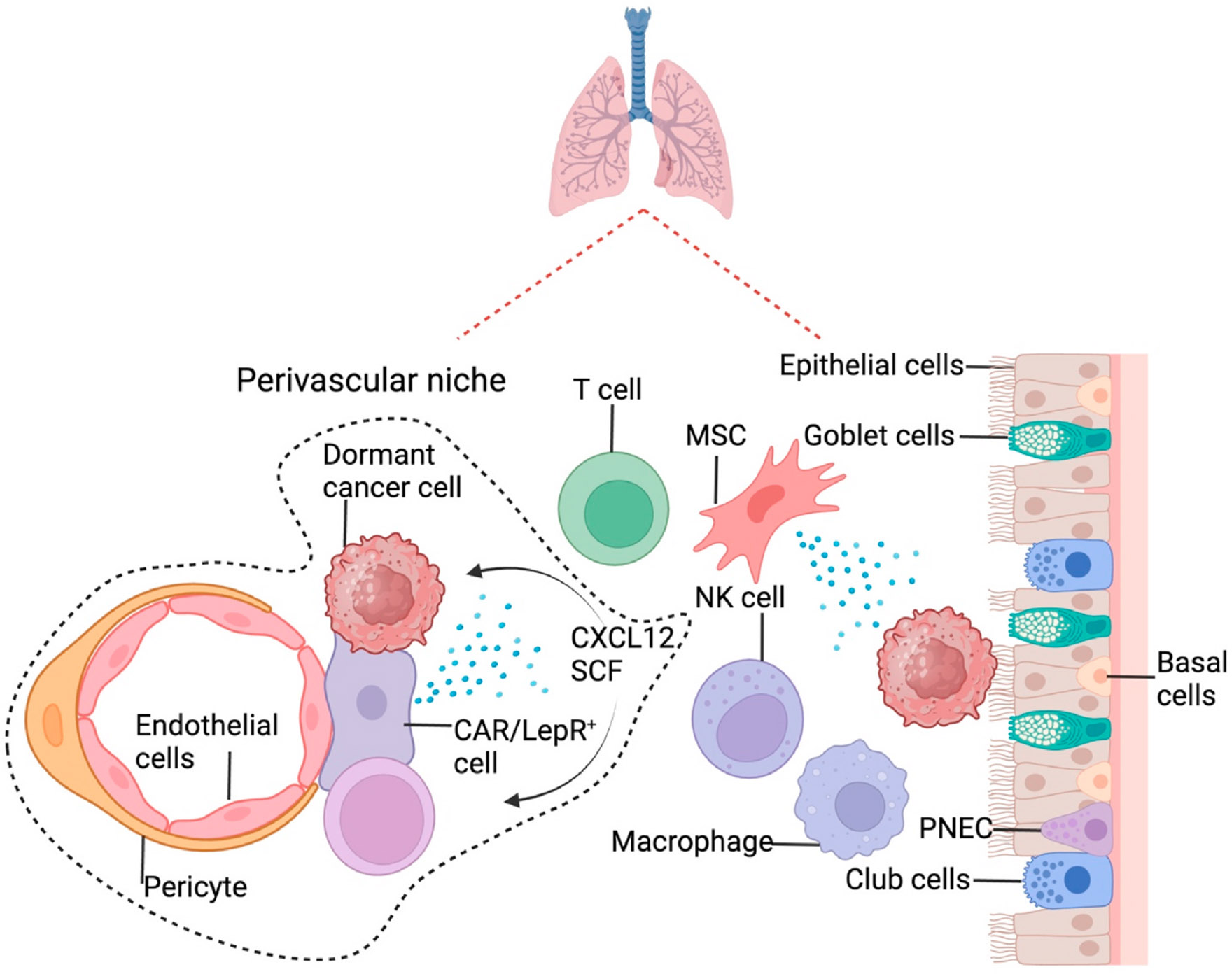
Dormancy niches in the lung. Endothelial cells, one of the essential components of the perivascular niches in the lung, are very important in keeping cancer cells dormant. Epithelial cells, especially alveolar epithelial type I cells, are key factors in the dormancy niches outside the perivascular region. HSC: Hematopoietic stem cell; CXCL12: CXC motif chemokine ligand 12; CAR cell: CXCL12-abundant reticular cell; LepR: Leptin receptor; SCF: Stem cell factor; MSC: Mesenchymal stem/stromal cell; NK cell: Natural killer cell; PNEC: Pulmonary neuroendocrine cells.

Furthermore, Ashani Weeraratna and colleagues compared the young and aged mice and found that the lungs in the young mice kept the melanoma cells dormant compared to the aged lungs. In the aged lung, the fibroblasts were reprogrammed to secrete sFRP1, inhibiting Wnt5A and AXL in melanoma cells and stimulating the outgrowth [69], suggesting a fibroblast-involved dormancy niche in the young lung for melanoma. Taken together, various dormancy niches exist in the lung (Fig. 4). However, whether the niches depend on cancer types or other conditions and how these niches regulate dormancy must be further delineated and pieced together.

### 4.4. Brain: the difference between early DTCs and the residual cancer cells after surgery debulking

**Fig. 5.**
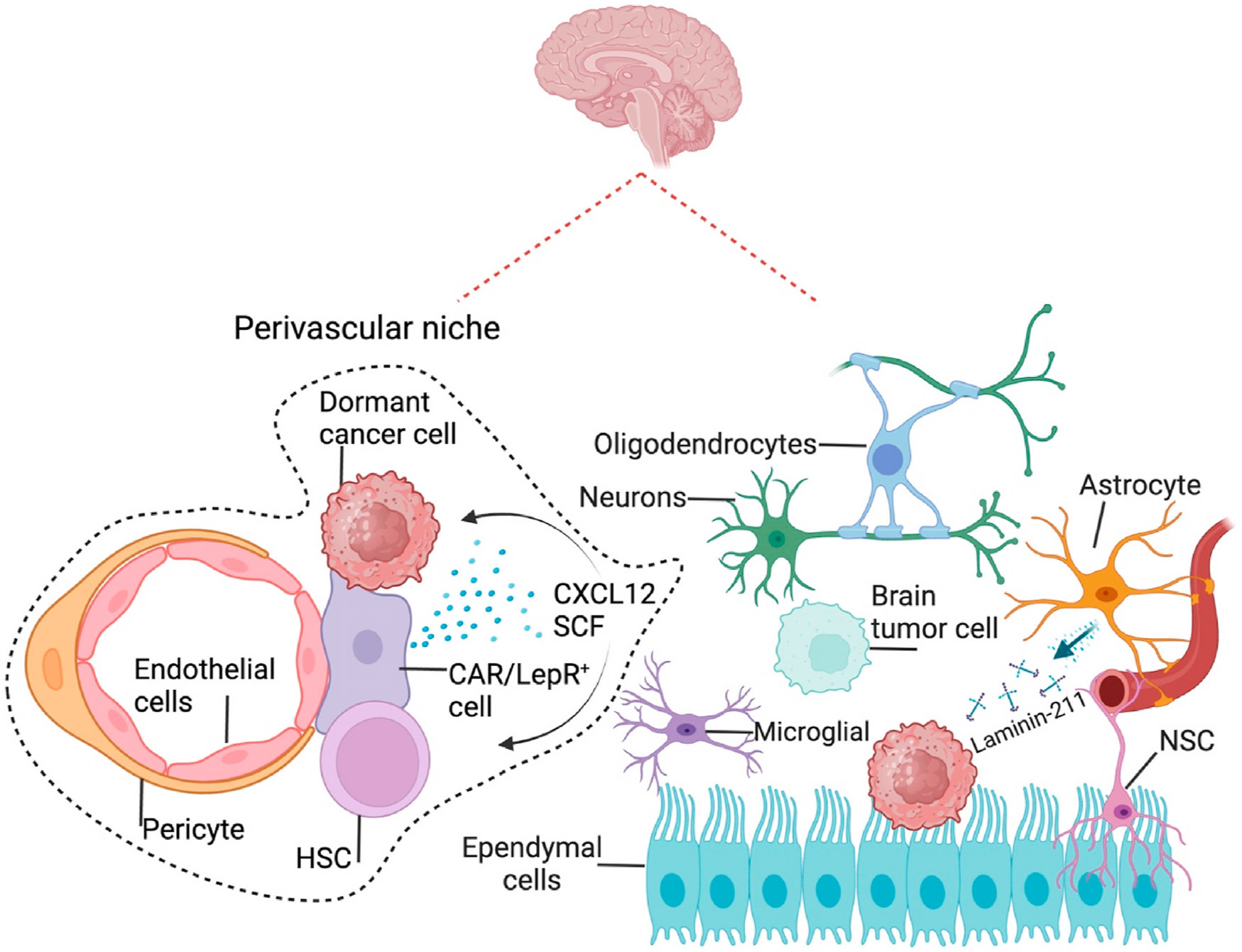
Dormancy niches in the brain. Currently, reported dormancy niches in the brain include the endothelial cells and pericytes in the perivascular areas, as well as the local astrocytes, which control the dormancy and outgrowth of cancer cells through secreted factors, such as laminin-211. HSC: Hematopoietic stem cell; CXCL12: CXC motif chemokine ligand 12; CAR cell: CXCL12-abundant reticular cell; LepR: Leptin receptor; SCF: Stem cell factor; MSC: Mesenchymal stem/stromal cell; NK cell: Natural killer cell; NSC: Neural stem cell.

Distinct from other organs, early DTCs are not the sole origin of dormant cancer cells in the brain. Metastatic cancer cells or brain tumor cells that were left at and within the surgery margins are also the sources that can later proliferate to cause brain tumor/metastasis relapse (Fig. 5). Consistent with hematogenous metastases, the endothelial cells and pericytes in the perivascular areas have also been demonstrated to constitute the dormancy/metastatic niches in brain tissues for BCa cells [48,125]. Furthermore, local brain cells, such as astrocytes, are reported to respond to the invaded BCa cells and provide a niche for solitary cancer cells’ survival, arrest, or metastatic outgrowth [126]. Astrocytes can deposit laminin-211, one of the key components of a dormancy niche for the disseminated BCa cells [127]. Specimens from the tumor/brain interface of patients who undergo neurosurgical resection of BCa brain metastases exhibited increased gliosis and infiltration of neural progenitor cells [126]. Moreover, the polarization of microglial cells has been found to facilitate brain metastasis of NSCLC [128]. Whether these local changes provide a dormancy niche for the dormant cells? An interesting study in glioblastoma showed that the bulk and marginal or infiltration tumor cells have distinct biology. The marginal tumor cells were mainly dormant glioblastoma cells and biased toward astrocyte-like differentiation. The dormant cells inside the bulk tumors resemble the features of the injured neural progenitor-like cells, such as the high expression of SRY-Box Transcription Factor 9 (SOX9) [129]. The bulk-derived dormant cells could reacquire the properties of Astro-like cells after exposure to the margin microenvironment, suggesting the essential role of the “niches,” which need to be further characterized.

### 4.5. Self-seeding: seeding not only to regional and distant sites but also returning to the primary site

The early DTCs, at least in part, account for the metastatic progression and recurrence [130]. The multi-directional seeding of the tumor cells, in addition to the distant sites reviewed above, are the tumors of origin. Self-seeding was first raised and published in 2006 by Dr. Norton and Massague based on logical thinking to unify cancer’s tumorigenesis versus metastatic behavior and biomathematics, i.e., fractal geometry and Gompertzian. The idea is that pathologic cell mobility, in addition to being crucial to invasion and metastasis, can also contribute significantly to primary tumor growth [131]. The same group provided evidence supporting this novel theory three years later, in which they observed the seeding of established tumors by circulating tumor cells (CTCs), which promoted primary tumor growth and stroma recruitment in breast, colon, and melanoma cancer cells. Thus, the large tumor grows from the “outside-in” instead of the “inside-out.” They revealed that the tumor-derived IL-6 and IL-8 attracted the CTCs, and the poor-prognosis markers, Matrix metalloproteinase 1 (MMP1), and fascin-1, mediated the CTC infiltration into the mammary tumors [132]. Self-seeding was further demonstrated and supported by other groups in and beyond BCa [132-138]. Furthermore, the “seeds” remained dormant for decades in such sites [53,139,140]. Under this theory, the primary tumors also provide a niche for dormant cells coming back “home” and act as a “sponge” for soaking up returning CTCs that contribute to locally advanced large tumors later, thus potentially sparing the development of lethal metastasis. Some tumor cells are more self-seeders, while others are more distant-seeders. This theory also helps explain why finding DTCs in distant organs does not always lead to detectable metastases, axillary nodal disease does not necessarily predict distant metastasis, and DTC absence does not guarantee no distant spread. It also sheds light on why surgical removal of additional lymph nodes does not reduce local/regional recurrence, while radiation therapy to the axilla may improve overall survival [53,141]. We refer to several reviews by Dr. Comen and Norton for detailed discussions of the critical questions on this topic [52,53].

Taken together, distant organs and primary tumors are all sites of dormancy niches. The organ-specific components at cellular, molecular, and ECM levels are to be further delineated for translational purposes, such as whether to keep the dormant niches to maintain cancer cells dormant to reach an “operational cure” or destroy the dormancy niches to eliminate the dormant cells that are the sources of metastatic progression and relapse. Notably, only major metastatic organs, such as the liver, lung, bone, and brain, have been frequently reported for dormancy niches. Efforts on the significance of dormancy niches in other organs or tissues such as lymph node [142] must be explored.

## 5. The secreted factors from the microenvironment regulate dormancy

Secreted factors are of interest as they might be detected in blood/serum as biomarkers for disease progression, prognosis, and treatment. To date, only a few secreted factors have been shown to have the capacity to induce dormancy. The ones with this role are context-dependent, organ-specific, and cancer type-specific. Together, these studies suggest the importance of the microenvironment where the DTCs reside. Direct cell-cell and cell-ECM interactions are likely the key.

### 5.1. Transforming growth factor-β (TGF-β)

Transforming growth factor-β (TGF-β) is a large family of secreted polypeptide growth factors comprising over 30 members, including activins, TGF-βs, and BMPs. Three distinct isoforms of TGF-β (TGF-β1/2/3) have been identified, and a different gene encodes each. TGF-β functions via interaction with cell surface receptors, the TGF type I, II, or III receptors (TGFBR) [143]. In normal physiology, TGF-β members regulate tissue homeostasis and regeneration. TGF-β, specifically TGF-β1, induces and maintains HSC quiescence through binding with the TGFBR2 and activating the downstream Smad 2/4 signaling pathway [144,145]. In cancers, the tumor promoter or suppressor role of TGF-β is context-dependent, spatially and temporally [146]. Since dormant PCa cancer cells were shown to compete with HSC for the HSC niches in the bones, it is not a total surprise that TGF-β involved in cancer dormancy. Interestingly, TGF-β2, through TGFBR1/3 and the downstream Smad 1/5, induced and maintained cancer dormancy in the mouse bone marrow of HNSCC [61], BCa [147], and PCa [35,45]. On the other hand, in the lungs, elevated levels of TGF-β1 were found in niches that stimulated disseminated BCa cell growth [48]. In an *in vitro* culture system, TGF-β1 induces the regrowth of dormant BCa MCF-7 cells [148].

Furthermore, TGF-β1 can stimulate cancer-associated fibroblasts (CAFs) to secrete interleukin-11 (IL-11), which enhances a high risk of colorectal cancer (CRC) relapse by triggering the GP130/STAT3 signaling in CRC cells [149]. CAFs, in turn, secreted TGF-β and other growth factors to create an immunosuppressive environment for cancer cells [150]. In non-small cell lung cancer (NSCLC), the intrinsic TGF-β/Smad 2 signaling in CAFs was elevated by taking the exosomes from cisplatin-induced dormant cancer cells; this, in turn, reduced the protection of CAFs to the cancer cells [151]. Clinically, NSCLC cells associated with this type of CAF are shown to be more sensitive to tyrosine kinase inhibitors [152].

Another growth factor involved in dormancy is fibroblast growth factor-2 (FGF-2), also known as basic FGF (bFGF). It can maintain mesenchymal stem cell (MSC) plasticity, stemness, and self-renewal and inhibit the senescence of MSCs [153-156]. In BCa, FGF2 was shown to induce cell cycle arrest in MCF-7 BCa cells, promote differentiation in T47-D and MDA-MB-231 cells, and induce cell dormancy through elevating integrin α_v_β_1_ [157-160]. However, these studies used an *in vitro* system, i.e., a fibronectin-coated cell culture plate. More studies are needed using different systems, including the most important *in vivo* system and correlation with patient data.

### 5.2. BMP4 and BMP7

Around 20 BMP family members have been identified and characterized. BMPs signal type I and II receptors, both indispensable for signal transduction, activating the downstream Smad1, 5, and 8. Three type I receptors are type IA and IB BMP receptors (BMPR-IA or Activin receptor-like kinase (ALK)-3 and BMPR-IB or ALK-6) and type IA activin receptor (ActR-IA or ALK-2) [161]. Three type II receptors are type II BMP receptor (BMPR-II) and type II and IIB activin receptors (ActR-II and ActR-IIB). In normal physiology, BMPs induce bone and cartilage formation and play a role in some non-osteogenic development processes [162]. For example, BMP-4 and 7 specifically induce a sympathetic adrenergic phenotype. In the clinic and dentistry, recombinant humanized BMP2 (rhBMP2) was used as a substitute/component for bone grafts in spinal surgery or dental implants [161]. Like TGF-β, which shows both positive and negative effects on tumorigenesis, BMPs also act as tumor promoters and suppressors in different types of cancer [163].

In PCa, rhBMP7 administration to nude mice inhibited the PCa metastases in intra-tibial and intra-cardiac injection models but not the orthotopic xenografted tumors [164]. Another study showed that BMP7 induced PCa dormancy through the BMP7-BMPR2-p38-N-Myc downstream-regulated gene 1 (NDRG1) axis in bone [62]. However, this study also showed that BMP7 stimulates the senescence of PCa stem-like cells, suggesting a broader effect of BMP7 than dormancy. In line with the role of BMP in promoting dormancy, Coco, a BMP/TGF-β/Wnt inhibitor [165], activated dormant metastatic murine BCa cells, whereas the knockdown of Coco induced dormancy in the lung. Mechanistically, Coco exerted this effect by blocking lung derived BMP4 ligands [63]. BMP4 was also shown to mediate glioma stem cell quiescence and subsequently confer treatment resistance [166].

### 5.3. The Wnt5a

The Wnt signaling pathway is an ancient and evolutionarily conserved pathway during development. The Wnts are nineteen proteins in humans [167]. Wnts bind with the Frizzled (Fz) receptor family of ten Fz to initiate the downstream canonical or Wnt/β-catenin dependent pathway and the non-canonical or β-catenin-independent pathway, which includes Planar Cell Polarity and the Wnt/Ca2+ pathways [167,168]. The non-canonical Wnts include Wnt2, 4, 5a, 5b, 6, 7b, and 11. They could bind unique co-receptors, such as receptor tyrosine kinase-like orphan receptor (ROR)1, 2, receptor-like tyrosine kinase (RYK), and protein tyrosine kinases (PTK)7 [167]. Wnts have an indispensable significant role in bone formation. Enhancing Wnt signaling, such as romosozumab, an anti-sclerostin antibody, was approved in 2019 by the Food and Drug Administration (FDA) for use in patients with a high risk of osteoporosis [169]. In cancer, however, most effects of Wnt signaling are tumor promoters or even drivers [170]. Although controversial data still need to be reconciled, the tumor-suppressing role of Wnt5a has been revealed in many types of cancer [171-180].

In terms of cancer dormancy, Wnt5a from osteoblastic niche induced dormancy of PCa cells in a reversible manner *in vitro* and *in vivo* through binding with ROR2, subsequently inducing Siah E3 ubiquitin protein ligase 2 expressions, which represses Wnt/β-catenin signaling [181]. Notably, a negative correlation of ROR2 expression with bone metastasis-free survival is observed in PCa patients. However, significant concerns were raised about the translational potential of this study if the *in vivo* studies were validated because the effects required 57- or 42-day consecutive treatment with rhWnt5a. On the other hand, aged lung fibroblasts increased sFRP1, which inhibits Wnt5a, thereby activating the dormancy of melanoma cells disseminated in the lung [69]. Furthermore, a recent study showed that Wnt5a mediates ROR2-dependent activation of the Hippo pathway to suppress YAP1/TAZ activity and tumor growth, supporting ROR2 expression and determining the role of Wnt5a in inhibiting cancer cell proliferation [182]. ROR2 may be a predictive marker for the opposite roles of Wnt5a observed. Furthermore, this study and others tested the effectiveness of Wnt5a-mimetic peptide drugs inhibiting tumor growth and metastasis, implying a better choice for future translation of manipulating Wnt5a [179,183-188].

Notably, secreted factors represent just one aspect of the tumor microenvironment (TME). Ideally, a secreted factor can serve as a prognosis marker if its level can be detected in any body fluid, such as serum. Based on current literature, we have yet to achieve this goal. Importantly, we all know that the surrounding TME is complicated; any component and influencer can contribute to cancer cells’ dormancy. For example, reduced nutrient availability, i.e., metabolic rewiring of dormancy in primary and metastatic tumors [189,190], low oxygen levels (hypoxia) [191,192], and immune surveillance [130,193]. The immune system plays a crucial role in controlling and maintaining the dormancy of cancer cells. Specific immune cells are critical for cancer dormancy in the TME. Most immune-related dormancy studies have been performed in blood cancers such as leukemia and lymphoma [12,194-205]. In solid tumors, one study showed that a single adoptive transfer of T cells killed CD11b^+^/Gr1^+^ myeloid cells in the tumor stroma and maintained the fibrosarcoma dormant [206]. Please refer to previous publications on exciting and important findings, thoughts, and debates on immunotherapy for dormant or active cancers [193,207-212].

## 6. Conclusion and perspective

Detections and early-stage treatments improve cancer patients’ overall survival and life quality. The major causes of cancer deaths are metastases and recurrence, and we still do not have better therapy or care for patients in these situations. We believe that the prevention of metastases and recurrence is the “cure” for cancer patients. Therefore, research in early dissemination and dormancy is essential. There are two ultimate goals to translate the research in this area. The first is to eradicate the early DTCs and dormant cancer cells. To reach this goal, drugs have been proposed and tested to reactivate the dormant cells into the cell cycle or sensitize therapy responses and combine them with respective therapies [15,213-215]. However, positive outcomes are yet to come in clinical trials. In addition, there are huge risks of reactivating every dormant cell and eliminating all of them. The ideal approach is to eradicate the dormant cells without reactivating them, which raises the challenges of tackling the wildly spread yet hard-to-detect DTC populations. How do we capture these minimal DTC populations? Proof-of-concept studies empowered direct targeting of the minimal residual DTCs and dormant cells once the specific markers were identified [216-219]. For example, the clinical study showed that applying a specific antibody, i.e., anti-VLA-4 (very late antigen-4), to the minimal residual disease of patients with acute myelogenous leukemia (AML) improved 5-year overall survival after chemotherapy [217]. However, the patient number is low. We believe future developments in this direction, including antibodies, vaccines, and chimeric antigen receptor (CAR) T cells, will benefit patients substantially. The second goal is maintaining dormancy since the dormant cells are asymptomatic, i.e., to reach an “operational cure,” which is popular and actively discussed in managing multiple myeloma [220]. Studies have been reported mainly on blocking the reactivation of dormancy to inhibit metastatic outgrowth [221]. Our recent publication tested a direct approach, i.e., drugs that induce dormancy and dormancy-mimicking [40], but the effectiveness in animal models needs to be further evaluated. We believe both approaches would work. Questions for the next steps are validation, drug development, and testing in preclinical settings.

This review focused on localized cellular and molecular changes; by no means is this a complete story of cancer dormancy. Systemic changes may also affect cancer dormancy. For instance, studies showed changes in psychological and stress hormones for lung and other types of cancers [222,223]. Whether and how do sex hormone changes, including changes through using hormonal therapies such as tamoxifen and enzalutamide, impact the dormancy of hormone-sensitive BCa and PCa [224,225]? We need to learn and be aware of all contributors and factors for the future success of translating our knowledge into the clinic.

## Figures and Tables

**Table 1:** Dormancy markers and regulations.

Markers	Cancer types	Models	Downstream mediators	Upstream regulators	Drugs (induce or reverse dormancy	references
uPAR (Down)	Epidermoid carcinoma (Hep3)	Culture, CAM, s.c.	α5β1/fibronectin, MEK1/ERK		PD98059 (MEK1 inhibitor), anti–uPAR and anti–β1 antibodies	[31-33,57-59]
P38α/β^High^ ERK^Low^	BCa, HNSCC, PCa, SCC, melanoma, fibrosarcoma	CM, s.c., cardiac injections, BM DTC, patient data & tumors	Transcription factor network (BHLHB3 or P53, c-Jun or FoxM1), DEC2/SHARP1, p21, p27, CDK4, NDRG1, SA-β-gal	TGFβ2/TGFBR3, uPAR, fibronectin, Cdc42, BMP7	TGFBRI inhibitors (LY-364947, SB431542), BMP inhibitor (Noggin, Coco)	[32,45,60-63]
NR2F1/DEC2	HNSCC, SACC, SCC, BCa, PCa, MMTV-Myc	s.c., tail vain, mammary fat pad injections. CAM. BM DTC from PCa (NED vs ADV), primary, recurrences and metastatic tumors from HNSCC	SOX9 and RARβ driven program, NANOG, Global repressive chromatin state, CXCL12/CXCR4.	H3K4me3 & H3K27ac, p38α, HER2, WNT4, NAS1	5-Aza-C and retinoic acid, CDK inhibitors, C26 (NR2F1 agonist)	[34,64-67]
TAM family of receptor tyrosine kinases, AXL or MERTK (Down)	PCa, melanoma	In vitro, intratibial injection, intradermally injected	EMT, MAP kinase signaling, p27, NR2F1, SOX2, NANOG	Hypoxia, sFRP1	CoCl2 SB203580	[35,37-39,68] [69]
ARHI	Ovary cancer,	In vitro Xenografting Patient data	Autophagic cell death, PI3K/AKT/mTOR, AMPK/TSC1/TSC2, p21, cyclin D1	Loss of heterozygosity	Chloroquie	[70,71]
Pfkfb3^Low^ Autophagy^High^	BCa	2&3D culture, mammary fat pad injection, patient data	p62/sequestosome-1	TGF-β1, Atg3, Atg7	Chloroquie	[72]
LIFR	BCa	Cell culture, intracardiac injection, patient data	Stat3, SOCS3	Hypoxia, HDAC, PTHrP	HDAC inhibitors, DMOG	[73,74]
Angiogenesis inhibitors such as thrombospondin 1 (TSP1), angiomotin, tropomyosin, EphA5, H2BK	BCa, osteosarcoma, glioblastoma, liposarcoma	2D &3D co-cultures, orthotopic injections, patient data	TGF-β1, 2, periostin, IGF binding protein	Ki67	TSP-1-blocking antibody, anti-integrin β_1_ or α_v_β_3_	[22,26-28,48,49]
mTOR and calcium pathways (Down)	BCa	Cell culture, Iliac injection, patient data	N- and E-adherins Connexin 43	S6K NFAT and MEF2	Anti-Ecad, Torin 1 or rapamycin, Danusertib and arsenic trioxide	[50,51]
HIF-1α	BCa, HNSCC, PDAC, CRC, MM, GBM	hypoxia-induction nanointravital devices, CAM, mouse models, patient sample	P27, C4orf47	P38, CSN8, FBX8, TRIM44, YAP/TAZ	CoCl2	[75-81]
Mitochondrial-related biological processes (Down)	PCa	2&3D co-culture with osteoblasts, patient data	NR2F1	FAK	PF-562,271 (FAK inhibitor)	[40]
Cell cycle arrest	PCa	Intro, serum deprivation	DNA synthesis	Akt/mTOR	Saikosaponin A	[82]

**Abbreviations:** ADV, Advanced Disease. ARHI, Aplasia Ras Homology Member I (also known as DIRAS3). AXL, AXL receptor tyrosine kinase. BCa, Breast Cancer. BM, Bone Marrow. C4orf47, centrosome-associated protein chromosome 4 open reading frame 47. CAM, Chick embryo chorioallantoic membrane. CM, Conditioned Media. CRC, colorectal cancer. CSN, COP9 signalosome. DMOG, Dimethyloxaloylglycine (a pharmacological activator of HIF signaling). DTC, Disseminated Tumor Cells. EphA5, Ephrin receptor A5. ERK, Extracellular signal-regulated Kinase. H2BK, Histone cluster 1 H2B family member K. HIF-1α, hypoxia-inducible factor-1α. HNSCC, Head-Neck Squamous Cell Carcinoma. FAK, focal adhesion kinase. FBX8, F-box protein 8. LIFR, Leukemia inhibitory factor receptor. MM, Multiple myeloma. NAS1, long non-coding RNA (IncRNA) NR2F1-AS1. NED, No Evidence of Disease. NDRG1, N-myc downstream-regulated gene 1. NR2F1 (COUP-TF1), Nuclear Receptor subfamily 2 Group F Member1. PDAC, Pancreatic ductal adenocarcinoma. Pfkfb3, 6-phosphofructo-2-kinase/fructose-2,6-biphosphatase 3. RA, Retinoic Acid. SACC, Salivary adenoid Cystic Carcinoma. SCC, Squamous Cell Carcinoma. S.C., subcutaneous xenografting in mice. SOCS3, Suppressor of cytokine signaling 3. TRIM44, tripartite motif containing 44. uPAR, urokinase plasminogen activator receptor.

## Abbreviations

ActR-I: Type IA activin receptors
ADV: Advanced Disease
Ajs: Adherens junctions
ALK-2: Activin receptor-like kinase-2
ALK-3: Activin receptor-like kinase-3
ALK-6: Activin receptor-like kinase-6
AML: Acute myelogenous leukemia
AMPK: AMP-activated protein kinase
AR: Androgen receptor
ARHI: Aplasia Ras Homology Member I (also known as DIRAS3)
Atg3: Autophagy related 3
Atg7: Autophagy related 7
BCa: Breast cancer
BHLHB3: Basic helix-loop-helix protein 3
BM: Bone marrow
BMP-1: Bone morphogenetic protein 1
BMP-4: Bone morphogenetic protein 4
BMP-7: Bone morphogenetic protein 7
BMPR: Bone morphogenetic receptor
CAFs: Cancer-associated fibroblasts
CAM: Chick embryo chorioallantoic membrane
CAR cell: CXCL12-abundant reticular cell
CAR T cell: Chimeric antigen receptor (CAR) T cell
CDK2: Cyclin-dependent kinase 2
CM: Conditioned Media
CRC: Colorectal cancer
CSN8: Cop9 signalosome subunit 8
CTC: Circulating tumor cells
CXCL12: CXC motif chemokine ligand 12
CXCR4: CXC motif chemokine receptor 4
DMOG: Dimethyloxaloylglycine
DTC: Disseminated tumor cell
ECM: Extracellular matrix
EMT: Epithelial–mesenchymal transition
EphA5: Ephrin receptor A5
ERK: Extracellular signal-regulated kinase
FAK: Focal adhesion kinase
FBX8: F-box protein 8
FDA: Food and Drug Administration
FGF-2: Fibroblast growth factor-2
FN: Fibronectin
Fz: Frizzled
GP130: Glycoprotein 130
HAN: Hyperplastic alveolar nodule
HDAC: Histone deacetylases
HER2: Human epidermal growth factor receptor 2
HIF-1α: hypoxia-inducible factor-1α
HNSCC: Head and neck squamous cell carcinoma
HSC: Hematopoietic stem cell
IGF: Insulin-like growth factor-1
IL-6: Interleukin-6
IL-8: Interleukin-8
IL-11: Interleukin-11
LepR: Leptin receptor
LIFR: Leukemia inhibitory factor receptor
MAPK: Mitogen-activated protein kinases
MEF2: Myocyte Enhancer Factor 2
MEK1: Mitogen-activated protein kinase kinase 1
MERTK: MER Proto-Oncogene, Tyrosine Kinase
MM: Multiple myeloma
MMP1: Matrix metalloproteinase 1
MMTV: Mouse mammary tumor virus
MSC: Mesenchymal stem cell
mTOR: Mammalian target of rapamycin
NAS1: NR2F1-AS1
NDRG1: N-myc downstream-regulated gene 1
NED: No Evidence of Disease
NFAT: Nuclear factor of activated T cells
NK cell: Natural killer cell
NKG2DL: NKG2D ligand
NPC: Non-parenchymal cell
NR2F1: Nuclear Receptor Subfamily 2 Group F Member 1
NSC: Neural stem cell
NSCLC: Non-small cell lung cancer
PCa: Prostate cancer
PDAC: Pancreatic ductal adenocarcinoma
PNEC: Pulmonary neuroendocrine cells
Pfkfb3: 6-phosphofructo-2-kinase/fructose-2,6-biphosphatase 3
PI3K: Phosphoinositide 3-kinase
PTHrP: Parathyroid hormone-related protein
RARβ: Retinoic Acid Receptor Beta
ROR: Receptor Tyrosine Kinase Like Orphan Receptor
RYK: Receptor-like tyrosine kinase
S6K: S6 kinase
SACC: Salivary adenoid Cystic Carcinoma
SCC: Squamous Cell Carcinoma
SCF: Stem cell factor
SFRP1: Secreted frizzled-related protein 1
SFRP2: Secreted frizzled-related protein 2
SOCS3: Suppressor of cytokine signaling 3
SOX2: SRY-Box Transcription Factor 2
SOX8: SRY-Box Transcription Factor 8
SOX9: SRY-Box Transcription Factor 9
STAT3: Signal transducer and activator of transcription 3
TAM family: Tyro3, Axl, and Mer
TGF-β: Transforming Growth Factor-β
TGFBR: Transforming Growth Factor-β receptor
TRIM44: Tripartite motif containing 44
TSC: Tuberous sclerosis
TSP1: Thrombospondin 1
uPA: Urokinase plasminogen activator
uPAR: Urokinase plasminogen activator receptor
